# Skeletal Muscle Apoptotic Signaling Predicts Thigh Muscle Volume and Gait Speed in Community-Dwelling Older Persons: An Exploratory Study

**DOI:** 10.1371/journal.pone.0032829

**Published:** 2012-02-28

**Authors:** Emanuele Marzetti, Hazel A. Lees, Todd M. Manini, Thomas W. Buford, Juan M. Aranda, Riccardo Calvani, Giorgio Capuani, Michael Marsiske, Donovan J. Lott, Krista Vandenborne, Roberto Bernabei, Marco Pahor, Christiaan Leeuwenburgh, Stephanie E. Wohlgemuth

**Affiliations:** 1 Department of Aging and Geriatric Research, Institute on Aging, University of Florida, Gainesville, Florida, United States of America; 2 Department of Orthopedics and Traumatology, Institute of Orthopedics, Catholic University of the Sacred Heart, Rome, Italy; 3 Department of Medicine, College of Medicine, University of Florida, Gainesville, Florida, United States of America; 4 National Research Council (CNR), Institute of Crystallography, Bari, Italy; 5 Department of Gerontology, Geriatrics and Physiatrics, Institute of Internal Medicine and Geriatrics, Catholic University of the Sacred Heart, Rome, Italy; 6 Department of Chemistry, “Sapienza” University of Rome, Rome, Italy; 7 Department of Clinical and Health Psychology, College of Public Health and Health Professions, University of Florida, Gainesville, Florida, United States of America; 8 Department of Physical Therapy, College of Public Health and Health Professions, University of Florida, Gainesville, Florida, United States of America; University of Valencia, Spain

## Abstract

**Background:**

Preclinical studies strongly suggest that accelerated apoptosis in skeletal myocytes may be involved in the pathogenesis of sarcopenia. However, evidence in humans is sparse. In the present study, we investigated whether apoptotic signaling in the skeletal muscle was associated with indices of muscle mass and function in older persons.

**Methodology/Principal Findings:**

Community-dwelling older adults were categorized into high-functioning (HF) or low-functioning (LF) groups according to their short physical performance battery (SPPB) summary score. Participants underwent an isokinetic knee extensor strength test and 3-dimensional magnetic resonance imaging of the thigh. Vastus lateralis muscle samples were obtained by percutaneous needle biopsy and assayed for the expression of a set of apoptotic signaling proteins. Age, sex, number of comorbid conditions and medications as well as knee extensor strength were not different between groups. HF participants displayed greater thigh muscle volume compared with LF persons. Multivariate partial least squares (PLS) regressions showed significant correlations between caspase-dependent apoptotic signaling proteins and the muscular percentage of thigh volume (R^2^ = 0.78; Q^2^ = 0.61) as well as gait speed (R^2^ = 0.81; Q^2^ = 0.56). Significant variables in the PLS model of percent muscle volume were active caspase-8, cleaved caspase-3, cytosolic cytochrome *c* and mitochondrial Bak. The regression model of gait speed was mainly described by cleaved caspase-3 and mitochondrial Bax and Bak. PLS predictive apoptotic variables did not differ between functional groups. No correlation was determined between apoptotic signaling proteins and muscle strength or quality (strength per unit volume).

**Conclusions/Significance:**

Data from this exploratory study show for the first time that apoptotic signaling is correlated with indices of muscle mass and function in a cohort of community-dwelling older persons. Future larger-scale studies are needed to corroborate these preliminary findings and determine if down-regulation of apoptotic signaling in skeletal myocytes will provide improvements in the muscle mass and functional status of older persons.

## Introduction

Over the past decades, the 75+ years age group has been the most rapidly expanding segment of the population in Western countries [1]. Unfortunately, this group is also the most susceptible to developing functional impairments and disability [2]. Indeed, the increase in life expectancy has not been paralleled by a proportional expansion in disability-free lifespan [3]. As a result, although several indices of functional limitations have shown improvements in the last two decades, over 20% of older American adults are presently disabled [3].

Sarcopenia, the age-related involuntary decline in skeletal muscle mass and function, is a major determinant of frailty and disability [4]. Furthermore, decreased muscle mass and strength are independently associated with mortality in older persons [5], [6]. The biological determinants of muscle aging remain elusive; however, several lines of evidence suggest that acceleration of apoptosis in skeletal myocytes during aging may represent a converging mechanism through which sarcopenia and physical function decline ensue (reviewed in [7]). Noteworthy, chronic low-grade inflammation and oxidative stress secondary to mitochondrial dysfunction, two processes believed to contribute to muscle aging [8], are powerful inducers of skeletal myocyte apoptosis [9], [10]. Moreover, down-regulation of apoptotic signaling in myocytes via pharmacological [11]–[14] and behavioral interventions [10], [15]–[19] or genetic manipulations [20] has been associated with attenuation of muscle loss and physical function decline in aged experimental animals. Conversely, up-regulation of apoptotic signaling secondary to the accumulation of critical levels of mitochondrial DNA (mtDNA) mutations, results in a premature aging phenotype and severe sarcopenia in mice expressing a proofreading deficient mtDNA polymerase [21]. Similarly, mice lacking the antioxidant enzyme copper/zinc-dependent superoxide dismutase (CuZnSOD or Sod1) develop marked sarcopenia accompanied by skeletal myocyte morphological and biochemical abnormalities, including enhanced apoptosis [22]. Remarkably, the genetic characterization of interleukin 10-deficient mice, a rodent model of frailty, unveiled up-regulation of several skeletal muscle apoptosis-related genes [23], further supporting the involvement of accelerated myocyte apoptosis in the pathogenesis of sarcopenia and physical frailty in late life.

Although studies in experimental animal models implicate apoptosis as a mechanism in muscle aging, evidence in humans is still lacking. To date, only three reports have been published examining the occurrence of skeletal myocyte apoptosis in older persons [24]–[26]. However, only one of these studies was performed on muscle specimens from living human subjects [26] and none investigated either specific biochemical pathways of apoptosis or the relationship between apoptotic signaling and measures of physical performance. Hence, the present exploratory study was designed to investigate whether the extent of apoptosis activation and signaling through specific apoptotic pathways were linked with functional status in older persons. The main hypothesis underlying this study was that older individuals with poor physical function would display reduced muscle mass and strength concomitant with enhanced apoptotic signaling relative to their high-functioning peers.

## Materials and Methods

### Ethics Statement

Prior to enrollment in the study, all participants provided written informed consent. In no case informed consent was obtained from next of kin, carers or guardians on the behalf of participants. The study was approved by the University of Florida's Institutional Review Board.

### Objectives

The aim of the study was to explore the relationship between the extent of apoptosis activation in the skeletal muscle and measures of muscle mass and physical performance in older persons. The relationship between signaling through specific apoptotic pathways and indices of muscle mass and function was also investigated.

### Participants

Participants were community-dwelling men and women aged 70 years or older, categorized in high-functioning (HF) or low-functioning (LF) based on their short physical performance battery (SPPB) summary score [27]. Specifically, persons with a performance score ≥11 were assigned to the HF group, whereas those with a summary score ≤7 were considered LF. These cutoff limits were selected based on their ability to predict several relevant health outcomes in older adults, including functional limitations, institutionalization and mortality [28]–[30]. Persons scoring 8–10 at the SPPB were excluded to allow a greater distinction in physical function and possibly muscle biochemical parameters between groups. Additional exclusion criteria were: smoking in prior 12 months; history of drug or alcohol abuse; engagement in regular physical exercise; active treatment for cancer or cancer in the past three years; heart failure New York Heart Association (NYHA) class III–IV; stroke with upper and/or lower extremity involvement; Parkinson's disease or other neurological disorders likely to interfere with physical function; major psychiatric illnesses; peripheral vascular disease Lériche-Fontaine stage 3–4; history of life-threatening cardiac arrhythmias; cognitive impairment (i.e., MiniMental State Examination score ≤21); renal disease requiring dialysis; lung disease requiring steroids; chronic viral diseases (e.g., hepatitis B and C, HIV); lower extremity amputation; severe knee or hip osteoarthritis limiting mobility; diabetes with visual, vascular or neuropathic complications; inflammatory diseases (e.g., rheumatoid arthritis, vasculitis, autoimmune disorders and inflammatory bowel disease); taking growth hormone, estrogen replacement, testosterone, anticoagulants, steroids, non-steroidal anti-inflammatory drugs on a regular basis; severe obesity [i.e., body mass index (BMI)≥35]; underweight (i.e., BMI≤18.5); active weight loss >5 kg in prior three months; lidocaine allergy; life-threatening illnesses with an estimated life expectancy <1 year. Temporary exclusion criteria were: recent bacterial/viral infection (<2 weeks); acute febrile illness in prior two months; high blood pressure (i.e., ≥180/110 mm Hg) at the screening visit; major surgery or hip/knee replacement in the past six months; statin treatment (subjects were asked to refrain from statin administration one month prior to the muscle biopsy upon their general practitioner's approval); other acute diseases interfering with mobility as indicated by the participant's general practitioner. Eligible persons were excluded if they had contraindications to the execution of magnetic resonance imaging (MRI), including claustrophobia, heart pacemaker/defibrillator, metallic stents, aneurysm clips, metal implants or prosthesis, neurostimulation systems, insulin pumps or other infusion pumps.

### Screening and recruitment procedures

Recruitment of participants was coordinated by the Recruitment Core of the University of Florida Claude D. Pepper Older Americans Independence Center. Recruitment strategies included media articles, direct mailings, newspaper announcements, and presentations to community groups. Following telephone screening, eligible persons were invited to attend a screening visit during which the purpose and procedures of the study were explained and informed consent was obtained. After the participant provided consent, a general assessment was completed to determine his/her standing height, body mass, BMI, and blood pressure.

### Description of Procedures and Investigations undertaken

#### Physical function assessment

To assess physical function, the SPPB and knee extensor strength were determined. The SPPB is composed of three subtasks: usual gait speed (GS), standing balance and chair-stand tests [27]. GS was evaluated over a 4-meter course at the person's usual pace. The faster of two trials (m/s) was used for the analysis. For the standing balance test, participants were asked to stand in three progressively more difficult positions for 10 s each: a side-by-side feet standing position, a semi-tandem position and a full-tandem position. For the chair-stand test, participants were asked to perform five repetitions of standing up and sitting down from a chair without using hands and the performance was timed. Each of the three SPPB subtasks was categorized into a 5-level score, with zero representing inability to do the test and four corresponding to the highest level of performance.

Knee extension strength was determined using a Biodex dynamometer (Biodex Medical System, Shirley, NY) to measure the maximal concentric isokinetic strength of knee extensors of the dominant leg. Participants were asked to exert their maximum force while extending the knee from 90° to 0° of flexion at 60° per second with a hip angle of 90–100°. Two practice repetitions were completed prior to three test repetitions. The maximal peak torque achieved was used for the analysis both as the absolute value (N⋅m) and the ratio between peak torque and BMI.

#### MRI for the quantification of thigh muscle volume

T1-weighted MRI was employed to quantify the thigh muscle volume (MV) of the dominant leg. Images were obtained using a 3.0-tesla magnet (Philips Medical Systems, Bothell, WA), as detailed elsewhere [31]. Briefly, three-dimensional data were collected using a body coil and a fast gradient-echo sequence, with repetition time (TR) = 100 ms, time to echo (TE) = 10 ms, flip angle = 30°, and chemically-selective fat suppression. Images were acquired with an encoding matrix of 256×256, a field of view of 16–24 cm and 7-mm slice thickness. For image analysis, the maximal cross-sectional area of the biceps femoris was identified. Subsequently, volumetric analysis was performed on 11 contiguous axial slices (10-mm thickness), five proximal and five distal from that corresponding to the maximal thigh cross-sectional area. Muscle tissue was quantified volumetrically, with results reported as the absolute volume (cm^3^) and percentage of the total thigh volume (hereby referred to as percent MV). The ratio between MV and BMI was also calculated.

Images were analyzed using the freely-available software package MIPAV 1.3 (Medical Image Processing, Analysis and Visualization; Center for Information Technology, National Institutes of Health, Bethesda, MD; http://mipav.cit.nih.gov). MRI data were also used for muscle quality determination, which was calculated as the ratio between maximal peak torque and thigh MV (N⋅m/cm^3^).

#### Muscle biopsy

Muscle samples were obtained from the vastus lateralis of the dominant leg by percutaneous needle biopsy, under local anesthesia [32]. Muscle specimens were cleaned of any visible blood and fat, frozen in liquid nitrogen and stored at −80°C until analysis.

#### Subcellular fractionation and immunoblotting

Isolation of cytosolic, mitochondrial and nuclear fractions from muscle samples was performed as previously described [9]. Electrophoresis and immunoblotting were carried out as detailed elsewhere [10]. Cytosolic fractions were used to assess the protein expression of cleaved caspase-3 (Millipore, Temecula, CA), active caspase-8 (Abcam, Cambridge, MA), tumor necrosis factor receptor 1 (TNF-R1; Abcam) and cytochrome *c* (Santa Cruz Biotechnology, Santa Cruz, CA). Expression levels of Bcl-2, Bax and Bak (all from Santa Cruz Biotechnology) were assayed in mitochondrial fractions. Finally, endonuclease G (EndoG; Abcam) and apoptosis-inducing factor (AIF; BD Biosciences, San Jose, CA) protein levels were assessed in mitochondrial and nuclear fractions. Digital images were captured with an Alpha Innotech Fluorchem SP imager (Alpha Innotech, San Leandro, CA) and analyzed using the built-in software as previously described [10].

### Statistical methods

Continuous descriptive variables were analyzed by the Mann-Whitney U test, whereas the χ^2^ test was used for categorical variables. To explore correlations between apoptotic signaling proteins and functional measures (i.e., GS, knee extensor strength and thigh muscle quality) or MRI data (percent MV), multivariate regressions were performed via partial least squares (PLS) analyses. Briefly, the idea beneath multivariate analysis methods, such as the PLS, is that each subject is represented by a single data point in a multidimensional space where each measured variable is one of the coordinate axes. The goal of multivariate PLS regressions is the reorientation of data points along new axes, that are algebraically expressed by a linear combination of the original variables, to guide the projections into meaningful directions by using an external (“response”) variable. The new axes are commonly referred to as “components” or “factors”.

PLS was performed on a data matrix where each subject was represented by a row, with each apoptosis-related variable corresponding to a column. Functional measures or imaging data were added as the variables against which multivariate regressions were run. Matrices were pre-processed by mean-centering and scaling (i.e., means of each column were set to zero and their standard deviations were set to one) [33]. This procedure is commonly applied to data matrices prior to PLS because it allows the comparison of covariations of variables independent of their numerical size, while maintaining their factorial structures [33]. PLS was validated by full cross-validation. According to this procedure, the same samples are used for both model estimation and testing. In each cross-validation step, one sample is left out from the calibration data set and the model is calibrated on the remaining data points. Values for the left-out samples are predicted, and prediction residuals are computed. The process is repeated with all other subsets of the calibration set until every object has been left out once. All prediction residuals are finally combined to compute the validation residual variance, commonly reported as “validation R^2^” or Q^2^. In addition, in order to assess the stability of validated models, the Marten's uncertainty test was applied, which couples the full cross-validation to the jackknife principle [34]. For every cross-validation sub-model, a set of model parameters (i.e., B-coefficients, scores, loadings and loading weights) were calculated, and variations over these sub-models estimated to assess the stability of results. The number of components on which PLS regression models were built was selected by the statistical software on the basis of the cross-validation test.

Separate PLS models were constructed for caspase-dependent (TNF-R1, active caspase-8, cleaved caspase-3, cytosolic cytochrome *c*) and caspase-independent apoptotic signaling proteins (mitochondrial and nuclear AIF, mitochondrial and nuclear EndoG), with mitochondrial Bcl-2, Bax and Bak included in both models. Apoptotic variables identified as significant by the uncertainty test were examined via Mann-Whitney U tests to determine differences between HF and LF subjects. The same test was used to compare PLS predictive apoptotic proteins between participants with percent MV or GS above and below the median value of the study sample. All tests were two-sided with significance set at p<0.05. All data are presented as mean ± standard deviation (SD).

PLS analyses were performed using the Unscrambler X 10.1 software (CAMO Software, Oslo, Norway), whereas the GraphPad Prism 4.0.3 software (GraphPad Software, San Diego, CA) was used for Mann-Whitney and χ^2^ tests.

## Results

### Descriptive characteristics of the study sample

A total of 43 community-dwelling older adults (25 HF and 18 LF) were recruited. Muscle biopsies yielded sufficient tissue to perform all biochemical analyses in 13 HF and 7 LF persons. Demographic, functional and imaging parameters as well as the number of comorbid conditions and medications in this subset of participants were consistent with the rest of the study sample, except for containing a higher proportion of males (75% vs. 35%; p<0.05). The main characteristics of participants with complete muscle biochemistry data are shown in Table 1. The two functional groups did not differ with respect to age, sex distribution, ethnicity, BMI or number of disease conditions and medications. Compared with LF persons, HF participants performed significantly better at the walking test and the chair-stand test of the SPPB [27]. In particular, the 4-meter walking speed, a functional parameter predictive of several relevant outcomes in older persons [35], was lower in the LF group relative to HF persons. No significant difference was determined between groups for the balance test score. However, HF participants displayed greater MV compared with the LF group. Finally, neither knee extensor strength nor thigh muscle quality (strength per unit volume) differed significantly between HF and LF persons.

**Table 1:** Characteristics of study participants with complete muscle biochemistry data according to the level of physical performance (high vs. low).

	HF (n = 13)n (%) or mean ± SD	LF (n = 7)n (%) or mean ± SD	p value
Age (years)	77.3±6.4	81.4±4.2	0.1651
Sex (female)	2 (15)	3 (43)	0.4169
Ethnicity			
- Caucasian	12 (92)	6 (86)	0.9012
- Afro-American	0 (0)	1 (14)	
- Other	1 (8)	0 (0)	
BMI (kg/m^2^)	27.2±3.6	27.6±3.8	0.8121
Number of comorbid conditions*	0.6±0.8	1.3±1.0	0.1075
Number of medications	2.2±1.4	2.4±1.5	0.6373
SPPB summary score	11.5±0.5	5.4±1.9	0.0004
- Balance test subscore	4.0±0.0	2.6±1.8	0.0545
- Chair-stand test subscore	3.5±0.5	0.6±0.8	0.0004
- 4-meter walking test subscore	4.0±0.0	2.3±0.5	0.0004
4-meter walking speed (m/s)	1.16±0.21	0.64±0.13	0.0006
Knee extensor strength			
- Absolute (N⋅m)	117.9±31.2	87.3±32.1	0.1131
- BMI-adjusted	4.34±1.25	3.20±1.21	0.0573
Thigh muscle volume			
- Absolute (cm^3^)	473.4±110.0	330.4±55.1	0.0044
- % of total thigh volume	52.4±8.5	37.2±8.2	0.0044
- BMI-adjusted	17.6±3.8	12.1±2.2	0.0056
Thigh muscle quality (N⋅m/cm^3^)	0.25±0.06	0.27±0.09	0.6920

*includes hypertension, coronary artery disease, prior stroke, peripheral vascular disease, diabetes, chronic obstructive pulmonary disease, and osteoarthritis.

### Multivariate PLS regressions of skeletal muscle apoptotic signaling proteins vs. imaging and functional data

**Figure 1:**
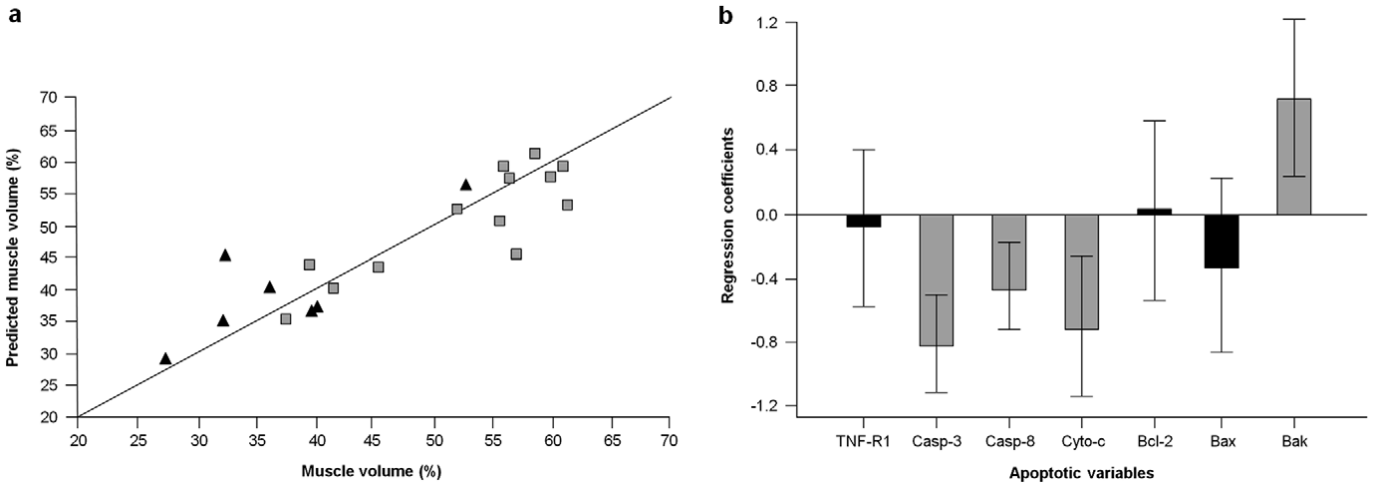
Multivariate PLS regression of caspase-dependent apoptotic signaling proteins versus percent muscle volume. (a) The regression yielded a 4-component model explaining 78.3% of the overall Y variance (Q^2^ = 0.61). Black triangles correspond to low-functioning participants; grey squares represent high-functioning subjects. (b) Regression coefficients of apoptotic signaling proteins according to the Marten's uncertainty test. Significant variables are represented by grey bars. Error bars indicate uncertainty limits.

**Figure 2:**
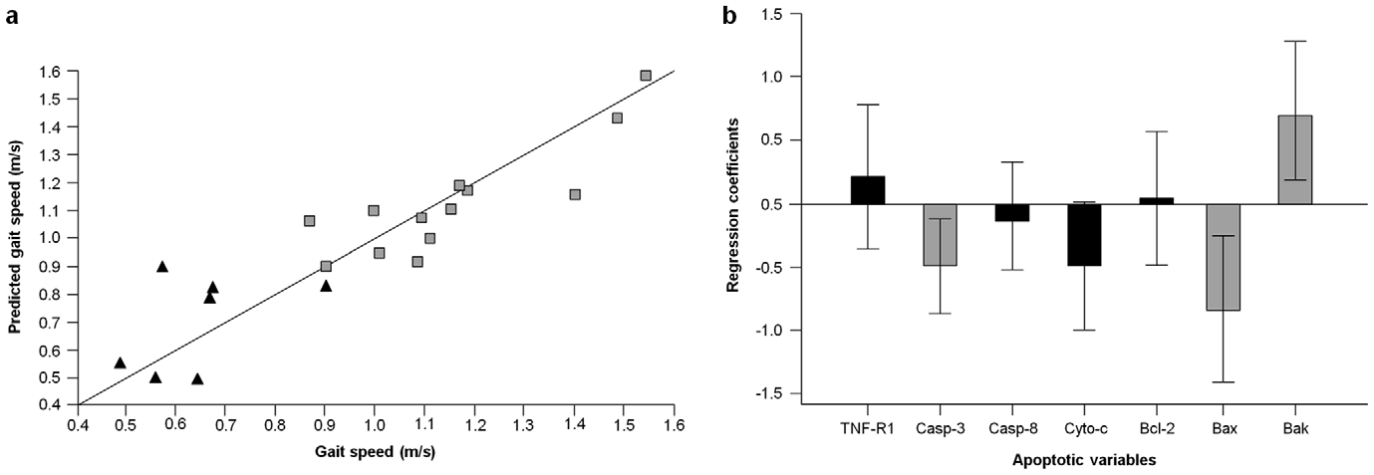
Multivariate PLS regression of caspase-dependent apoptotic signaling proteins versus gait speed. (a) The regression yielded a 4-component model explaining 81.3% of the overall Y variance (Q^2^ = 0.56). Black triangles correspond to low-functioning participants; grey squares represent high-functioning subjects. (b) Regression coefficients of apoptotic signaling proteins according to the Marten's uncertainty test. Significant variables are represented by grey bars. Error bars indicate uncertainty limits.

The regression of caspase-dependent apoptotic signaling proteins versus percent MV yielded a regression model based on four components, explaining 78.3% of the overall Y variance, with a cross-validation Q^2^ of 0.61 (Figure 1A). The uncertainty test revealed that four of the original variables were mainly involved in the description of the model, namely active caspase-8, cleaved caspase-3 and cytosolic cytochrome *c* (inverse correlation), and mitochondrial Bak (direct correlation) (Figure 1B). No correlation was determined between caspase-independent signaling proteins and percent MV (data not shown). Moreover, neither caspase-dependent nor caspase-independent apoptotic signaling proteins were correlated with knee extensor strength or thigh muscle quality (data not shown). A 4-component model was obtained from the regression of caspase-dependent apoptotic signaling proteins versus GS. The model explained 81.3% of the overall Y variance, with a cross-validation Q^2^ of 0.56 (Figure 2A). Variables involved in the description of this PLS model were cleaved caspase-3 and Bax (inverse correlation), and Bak (direct correlation) (Figure 2B). Caspase-independent apoptotic signaling proteins were not correlated with GS (data not shown).

**Figure 3:**
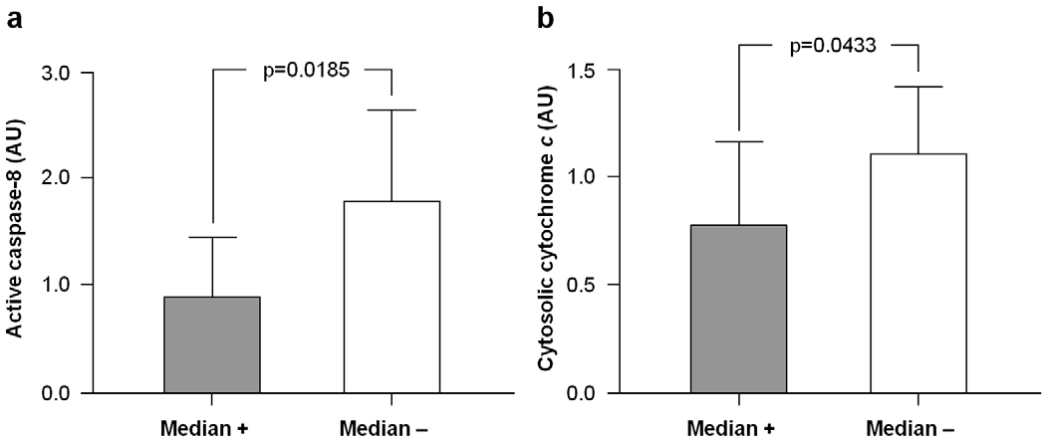
Protein expression levels of active caspase-8 and cytosolic cytochrome *c* according to the median of percent muscle volume. The content of active caspase-8 (a) and cytosolic cytochrome *c* (b) was higher in participants with percent muscle volume below (median −) the median value of the study sample (48.6%) relative to those with percent muscle volume above the median (median +). AU: arbitrary units.

Comparisons of PLS predictive apoptotic variables between HF and LF participants via Mann-Whitney U tests revealed no significant differences (Table 2). However, protein levels of active caspase-8 and cytosolic cytochrome *c* were higher in persons with percent MV below the median value of the study sample (48.6%; LF = 6, HF = 4; Figure 3). Conversely, levels of apoptotic proteins predictive of GS were not significantly different in participants walking faster than the median speed of the study population (1.01 m/sec; LF = 0, HF = 10) compared with slower walkers (LF = 7, HF = 3; data not shown).

**Table 2:** Expression levels of PLS predictive apoptotic signaling proteins in the two functional groups.

	HF (n = 13)Mean ± SD	LF (n = 7)Mean ± SD	p value
Cleaved caspase-3	1.20±0.32	1.56±0.61	0.2346
Active caspase-8	1.46±0.92	1.08±0.61	0.4758
Mitochondrial Bak	0.99±0.52	0.85±0.24	0.8741
Mitochondrial Bax	0.70±0.36	0.80±0.23	0.3417
Cytosolic cytochrome *c*	0.86±0.41	1.01±0.34	0.4281

Data are expressed in arbitrary optical density units.

In summary, multivariate PLS regression analyses identified significant correlations between caspase-dependent apoptotic signaling proteins and percent MV as well as GS, in the absence of clear differences in individual apoptotic signaling proteins between the two functional groups.

## Discussion

Preclinical studies have implicated apoptosis in skeletal myocytes as a mechanism contributing to sarcopenia. Whether apoptotic signaling is associated with muscle mass and function in older adults has yet to be established. Here, we investigated the relationship between apoptotic signaling and measures of muscle mass and physical performance in a cohort of relatively healthy, community-dwelling older persons. Our analyses indicate that signaling proteins pertaining to caspase-dependent apoptotic pathways are predictive of percent MV and GS. Conversely, no significant correlations were determined between caspase-independent apoptotic signaling proteins and imaging or functional measures. Finally, none of the apoptotic pathways investigated was correlated with either knee extensor strength or thigh muscle quality. Noteworthy, our results indicate that caspase-dependent apoptotic signaling proteins are predictive of two parameters (i.e., percent MV and GS) that have recently been proposed by the European Working Group on Sarcopenia in Older People (EWGSOP) for the screening and diagnosis of sarcopenia in clinical settings [4].

The loss of muscle mass and function with advancing age is a major determinant of frailty, disability and mortality [4]. Hence, the identification of biological pathways underlying muscle aging is of utmost importance for designing targeted interventions against a major health issue in the elderly. Accumulating preclinical evidence suggests that an acceleration of apoptosis in skeletal myocytes occurs over the course of aging, likely contributing to the pathogenesis of sarcopenia (reviewed in [7]). Findings from the present study are consistent with these observations and demonstrate for the first time that apoptotic signaling is correlated with a measure of muscle mass in older persons. In keeping with our previous studies in laboratory rodents [9], [10], [18], our findings also suggest that signaling proteins of the death-receptor and mitochondria-mediated apoptotic pathways may be involved in the pathogenesis of human muscle aging. Indeed, caspase-8 becomes engaged in the apoptotic signaling following binding of TNF-α to its cell surface receptor, and subsequently activates effector caspases, such as caspase-3 (death-receptor apoptotic pathway) [36]. Furthermore, through the cleavage of pro-apoptotic Bid, caspase-8 represents a point of convergence between TNF-α signaling, mitochondria-driven apoptosis and mitochondrial dysfunction [37]. Hence, our data may provide a further explanation to previous reports showing that elevated circulating TNF-α levels are associated with reduced muscle mass in advanced age (reviewed in [38]). Moreover, our results are consistent with the involvement of mitochondrial apoptotic signaling in muscle loss in old age. Indeed, mitochondrial Bak and cytosolic cytochrome *c* were among the variables mainly involved in the description of the regression between apoptotic signaling and percent MV (Figure 1B). It is interesting to note that, while cytosolic cytochrome *c* levels were inversely correlated with percent MV, which is consistent with the pro-apoptotic activity of this molecule upon its release from mitochondria, a direct correlation was determined for Bak. According to the current understanding, Bak, in association with other apoptogenic proteins (e.g., Bax), forms of a pore in the outer mitochondrial membrane, through which apoptotic factors such as cytochrome *c* can be released from the mitochondrial intermembrane compartment into the cytoplasm [39]. One possible explanation to the discrepancy between our findings and the pro-apoptotic role traditionally attributed to Bak arises from the demonstration of the existence of an anti-apoptotic Bak isoform (N-Bak) generated through alternative splicing [40]. Although N-Bak appears to be neuron-specific, the possibility that a similar anti-apoptotic Bak variant might also be expressed in the skeletal muscle warrants further investigation.

Several mechanisms may be invoked to explain the relationship between myocyte apoptosis and muscle loss. In multinucleated skeletal myofibers, the execution of apoptosis results in the elimination of individual myonuclei (myonuclear apoptosis) and a corresponding portion of the sarcoplasm. Over time, this process, coupled with insufficient satellite cell replenishment, may eventually lead to fiber atrophy [41]. Furthermore, apoptotic signaling may induce the degradation of contractile proteins, resulting in decreased myofiber cross-sectional area without myonuclear removal or fiber death [42]. In fact, caspase-3 is required for initiating the proteolytic degradation of actinomyosin complexes and myofibrils by generating monomeric actin and actin fragments which are subsequently degraded by the proteasome [43].

Another major finding of the study is that apoptotic signaling in the skeletal muscle is predictive of walking speed. In recent years, GS at usual pace has been increasingly recognized as a powerful predictor of adverse health outcomes in older persons, including disability, hospitalization, institutionalization and mortality [28], [30], [44]. As such, GS is being advocated as an additional vital sign in older adults, due to its potential to distinguish chronological from biological age [35]. Although walking speed is dependent on multiple organ systems (e.g., cardiovascular, respiratory and nervous systems), it is undoubted that muscle function is central in determining how fast an individual can walk. From this perspective, the correlation between apoptotic signaling proteins and GS observed in our study is especially intriguing, as it suggests that this cellular pathway may be involved in the disabling process. The mechanisms whereby apoptosis may impact GS are multifaceted. As previously discussed with regard to muscle atrophy, apoptotic signaling in skeletal myocytes may result in the degradation of contractile elements, followed by reduced muscle mass and force generation with or without myonuclear elimination. In addition, apoptotic signaling may be accompanied by mitochondrial bioenergetic failure and increased generation of reactive oxygen species (ROS), resulting in ATP depletion and oxidative damage to cellular macromolecules [45]. This hypothesis is supported by the observation that impaired mitochondrial function precedes the initiation of apoptosis in skeletal myocytes of laboratory rodents [46], [47]. Noteworthy, in our study sample, mitochondrial Bax and Bak were among the variables describing the regression model between apoptotic signaling proteins and GS (Figure 2B). Furthermore, the regression coefficient of cytosolic cytochrome *c* was close to the statistical significance. These findings suggest that mitochondrial apoptotic signaling, and potentially mitochondrial dysfunction, may contribute to decreasing physical function at old age.

None of the apoptotic signaling pathways investigated correlated with either muscle strength or quality. Moreover, these functional measures did not differ significantly between HF and LF participants. Indeed, muscle strength was not correlated with either the SPPB summary score or GS (data not shown). These findings may be explained by the small sample size analyzed and possibly by technical difficulties in achieving a true maximal peak torque. Reasons underlying such limitation include a lack of confidence of older persons with physical testing and apprehension toward unfamiliar laboratory devices [48], [49]. In addition, the possibility exists that different levels of co-contraction could have occurred in the two groups, thus influencing the strength testing results. Finally, among PLS variables correlated with percent MV, only active caspase-8 and cytosolic cytochrome *c* differed significantly between participants with MV above or below the median value of the study sample (Figure 3). In the case of GS, none of the PLS significant variables differed between median groups. Similarly, no differences in the apoptotic variables describing PLS models were observed between functional groups. While the discrepancy between PLS regressions and univariate tests may be related to the small sample size analyzed, an alternative explanation for this divergence can be proposed. Indeed, the impact of apoptosis on functional and imaging parameters is likely the result of the coordinated actions of multiple apoptotic signaling proteins, rather than reflecting the effect of individual mediators. In this scenario, multivariate analyses, such as PLS regressions, may be better suited to capture the information resulting from the interaction among multiple signaling proteins.

In conclusion, findings from this study indicate that apoptotic signaling in the skeletal muscle is correlated with percent MV and GS in a cohort of relatively healthy, community-dwelling older persons. Our data also suggest that specific signaling pathways of apoptosis (i.e., mitochondrial caspase-dependent and death-receptor pathways) are linked with measures of muscle mass and physical function. These findings confirm previous observations in animal models and suggest a novel biological target for interventions against muscle aging and physical function decline. Future larger-scale studies are needed to substantiate these preliminary results and determine if down-regulation of apoptotic signaling in skeletal myocytes via behavioral or pharmacological interventions will provide improvements in the muscle mass and functional status of older persons.

### Limitations

Although reporting novel findings, the present work presents some limitations that deserve further discussion. First, the study is of exploratory nature, evident by the small sample size. This might have hindered the detection of significant correlations between apoptotic signaling proteins and muscle strength as well as the observation of differences between functional and median groups in the apoptotic variables describing PLS models. Moreover, considering that skeletal myocyte apoptosis occurs throughout the lifespan and supposedly accelerates in late life, only a “snapshot” of apoptotic signaling could be detected. Despite this limitation, the present data show that a set of apoptotic signaling proteins correlates with imaging and functional parameters, which makes our findings highly relevant. It should also be considered that recruiting LF older persons without severe comorbidity poses a significant challenge, which adds further value to our results. Circulating levels of inflammatory biomarkers associated with physical performance (e.g., C-reactive protein, TNF-α, interleukin 6) were not measured. Therefore, correlations between inflammatory markers and apoptotic signaling proteins could not be explored. Although only subjects not engaged in regular exercise were enrolled, the amount of physical activity of participants was not quantified. Hence, the relationship between apoptotic signaling and the overall level of physical activity could not be established. In addition, due to its cross-sectional design, this study does not allow to infer causality between apoptotic signaling and measures of muscle mass and physical function. Future investigations will have to determine if the mitigation of apoptosis through behavioral or pharmacological interventions results in amelioration of sarcopenia and physical performance. Finally, due to tissue limitation, biochemical analyses were restricted to key components of specific apoptotic pathways and the extent of DNA fragmentation could not be assessed. However, the quantification of cleaved caspase-3 expression is considered to be a reliable marker of apoptosis [50].
